# Examining Usability, Acceptability, and Adoption of a Self-Directed, Technology-Based Intervention for Upper Limb Rehabilitation After Stroke: Cohort Study

**DOI:** 10.2196/45993

**Published:** 2023-08-21

**Authors:** Michelle Broderick, Robert O'Shea, Jane Burridge, Sara Demain, Louise Johnson, Paul Bentley

**Affiliations:** 1 Department of Brain Sciences Imperial College London London United Kingdom; 2 Department of Cancer Imaging Kings College London London United Kingdom; 3 School of Life Sciences University of Southampton Southampton United Kingdom; 4 University Hospitals Dorset NHS Foundation Trust Bournemouth United Kingdom

**Keywords:** stroke rehabilitation, interactive gaming, rehabilitation technology, technology usability, technology acceptability, self-management, usability, acceptability, stroke, rehabilitation, adoption, engagement, acceptance, limb, mobility, mobile phone

## Abstract

**Background:**

Upper limb (UL) recovery after stroke is strongly dependent upon rehabilitation dose. Rehabilitation technologies present pragmatic solutions to dose enhancement, complementing therapeutic activity within conventional rehabilitation, connecting clinicians with patients remotely, and empowering patients to drive their own recovery. To date, rehabilitation technologies have been poorly adopted. Understanding the barriers to adoption may shape strategies to enhance technology use and therefore increase rehabilitation dose, thus optimizing recovery potential.

**Objective:**

We examined the usability, acceptability, and adoption of a self-directed, exercise-gaming technology within a heterogeneous stroke survivor cohort and investigated how stroke survivor characteristics, technology usability, and attitudes toward technology influenced adoption.

**Methods:**

A feasibility study of a novel exercise-gaming technology for self-directed UL rehabilitation in early subacute stroke survivors (N=30) was conducted in an inpatient, acute hospital setting. Demographic and clinical characteristics were recorded; participants’ performance in using the system (usability) was assessed using a 4-point performance rating scale (adapted from the Barthel index), and adherence with the system was electronically logged throughout the trial. The technology acceptance model was used to formulate a survey examining the acceptability of the system. Spearman rank correlations were used to examine associations between participant characteristics, user performance (usability), end-point technology acceptance, and intervention adherence (adoption).

**Results:**

The technology was usable for 87% (n=26) of participants, and the overall technology acceptance rating was 68% (95% CI 56%-79%). Participants trained with the device for a median of 26 (IQR 16-31) minutes daily over an enrollment period of 8 (IQR 5-14) days. Technology adoption positively correlated with user performance (usability) (ρ=0.55; 95% CI 0.23-0.75; *P*=.007) and acceptability as well as domains of perceived usefulness (ρ=0.42; 95% CI 0.09-0.68; *P*=.03) and perceived ease of use (ρ=0.46; 95% CI 0.10-0.74; *P*=.02). Technology acceptance decreased with increased global stroke severity (ρ=−0.56; 95% CI −0.79 to −0.22; *P*=.007).

**Conclusions:**

This technology was usable and acceptable for the majority of the cohort, who achieved an intervention dose with technology-facilitated, self-directed UL training that exceeded conventional care norms. Technology usability and acceptability were determinants of adoption and appear to be mediated by stroke severity. The results demonstrate the importance of selecting technologies for stroke survivors on the basis of individual needs and abilities, as well as optimizing the accessibility of technologies for the target user group. Facilitating changes in stroke survivors’ beliefs and attitudes toward rehabilitation technologies may enhance adoption. Further work is needed to understand how technology can be optimized to benefit those with more severe stroke.

## Introduction

Stroke rehabilitation outcomes are strongly influenced by dose, or amount, of rehabilitation [1-5]. Rehabilitation dose in conventional clinical practice is insufficient for meaningful improvements in upper limb (UL) outcomes [6]. Increasing dose presents organizational and individual challenges [7,8]; digital technologies may offer a solution to this [9-13]. Technologies have the potential to complement therapeutic activity within conventional rehabilitation, connect clinicians with patients remotely, and empower patients to drive their own recovery, reducing the burden on rehabilitation services, overcoming regional resource disparities, and increasing access to rehabilitation [14].

Rehabilitation technologies often encompass behavior change concepts, which serve to optimize user engagement (goals and planning, feedback and monitoring, repetition and substitution, comparison of outcomes, reward and threat) [15], as well as features and components that enhance conditions for motor relearning [16]. These features include enriched environments, multisensorial stimulation, opportunities for massed practice that is variable, task-specific, and goal-oriented, real-time and longitudinal performance feedback, results feedback, increasing difficulty, and adjusting to each user’s unique and changing needs or abilities. In this work, we focus on self-directed rehabilitation technologies that enable users to complete >50% of training independently [17], allowing for formal or informal support for intervention components such as obtaining and setting up equipment and charging electrical devices. These interventions are of particular interest in the current health care context, due to the potential resource efficiency; bolstering the ability of stroke survivors to engage in rehabilitation activities with minimal professional support and thus presenting a pragmatic solution to dose enhancement and facilitating increased access to rehabilitation across the stroke recovery pathway.

While rehabilitation technology research has become increasingly prevalent in line with technological innovations in this field [18], clinical adoption remains poor [19,20]. Perceived barriers and facilitators to the adoption of stroke rehabilitation technologies have been proposed [20-31], influencing technology design in terms of accessibility, reliability, adaptability, and clinical utility [23,27,29,32-38]. Previous research focuses on design features of the devices, whereas the influence of stroke survivor characteristics, the usability of technologies, and users’ attitudes and beliefs about rehabilitation technologies are poorly understood [39], limiting clinical interpretation and generalizability [40]. Moreover, most previous studies of technology adoption are based on research environments with high levels of support and supervision rather than on unsupervised, natural environments, where stroke survivors spend the majority of their time [41-44].

**Figure 1:**
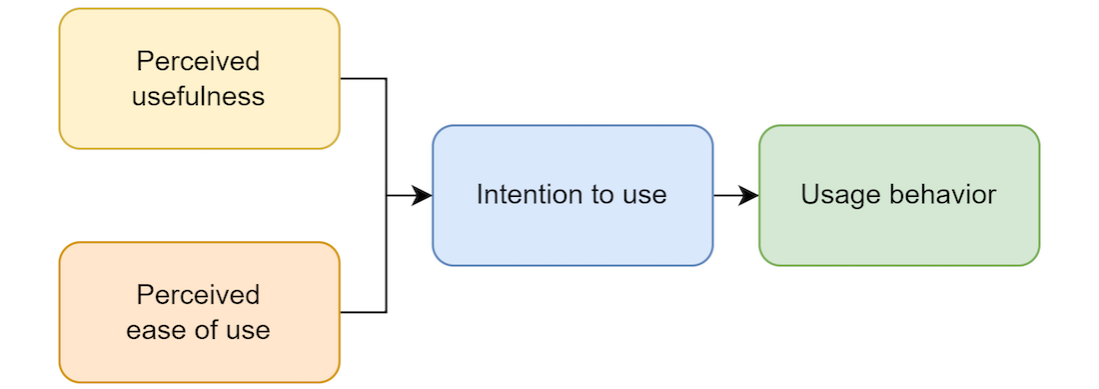
Technology acceptance model [49].

Technology usability (or user performance) refers to a measure of how well a specific user, in a specific context, can use technology to achieve a defined goal effectively and efficiently [45]. Usability is a key theme presented in qualitative literature examining the perceptions of stroke survivors and clinicians and their experiences of rehabilitation technologies [26,46]. Usability is also central in the design of rehabilitation technologies; however, usability outcomes are rarely reported in clinical trials [45]. Technology acceptance refers to the user’s willingness to use technology for its intended use. It is widely considered as a preadoption stage and also has value in predicting adoption [47]. Like usability, technology acceptability is thought to be associated with specific stroke survivor characteristics including age, sex, previous experience with technology, available support, and time since stroke [48]. The technology acceptance model (TAM) [49] proposes that acceptability is determined by 2 main factors: perceived ease of use and perceived usefulness [49]. Perceived ease of use refers to the degree to which a person believes that the use of a system will be effortless, while perceived usefulness refers to the degree to which a person believes that the use of a system will be advantageous to them [49]. The easier the use of a system is perceived to be, the higher the probability that a person experiences the system as useful and subsequently is willing to use it [49] (Figure 1).

The TAM has been frequently adapted to understand the acceptance of health care technologies among clinicians [50-55]. Perceived ease of use and perceived usefulness have been strongly associated with the adoption of telemedicine platforms in a stroke context [56]. Different factors are reported as important in predicting technology acceptance among different professional stakeholders [56], for example, in telemedicine trials, perceived ease of use was found to be more important to nonnurses (radiologists, physicians, and allied health care professionals) and perceived usefulness was more important to nurses. Perceived usefulness of telemedicine services is a major factor explaining adoption by clinicians [57]. Only a small number of studies [58,59] have applied the TAM to examine stroke survivors’ acceptance of UL rehabilitation technology (interactive gaming and mobile rehabilitation apps); however, these studies do not evaluate real-world adoption or consider stroke survivor characteristics. This study evaluates how real-world adoption, in the absence of close professional support, relates to acceptance, usability, and participant characteristics.

## Methods

### Ethics Approval

The study was approved by the UK National Research Ethics Service (78462). All participants gave informed written consent prior to recruitment.

### Study Design

This paper reports the results of a questionnaire survey of stroke survivors enrolled in a prospective, nonrandomized feasibility study of an adapted UL rehabilitation system for self-directed rehabilitation.

### Aim

The aim of the study is to explore the usability, acceptability, and adoption of a low-cost, self-directed, exercise-gaming technology while examining the impact of relevant user demographics and clinical variables in a heterogeneous stroke survivor cohort (see Multimedia Appendix 1 for a diagrammatic representation of this working theory or hypothesis in the form of a logic model). Research feasibility results are discussed in a separate publication [60].

### Patient Population

**Figure 2:**
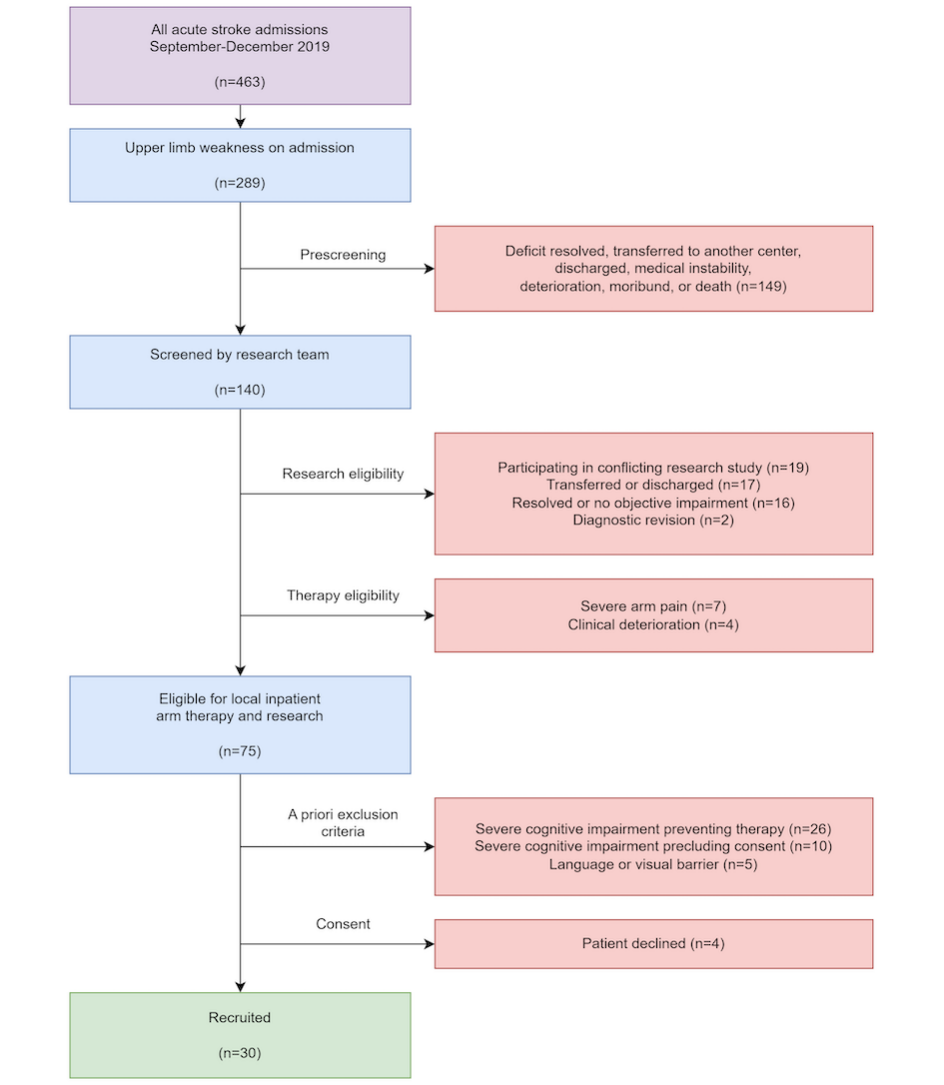
Recruitment flow diagram.

Participants were a convenience sample of inpatient, early subacute stroke survivors (n=30) in hyperacute or acute stroke units at a single center, presenting with new UL weakness (of any severity) and able to provide informed consent. Those with uncompensated visual deficits, unremitting UL pain, or significant language or communication difficulties were excluded. Patients were screened and referred by the treating clinical team at a central London stroke center (turnover ~1500 stroke cases per annum) between September and December 2019 (Figure 2).

### Intervention

**Figure 3:**
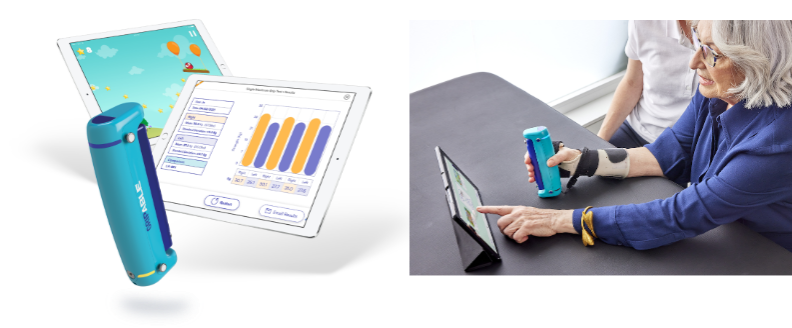
GripAble device and patient using device. The image demonstrates the patient performing single-player grasp and release activity. Images copyright of GripAble.co, reused with permission.

An interactive exercise-gaming system (nonimmersive virtual reality) [17,61] aimed at improving UL motor recovery after stroke by promoting self-directed, repetitive UL activity was used. The technology comprised a flexible, handheld device that sensed grip force as well as tracking finger, wrist, and arm movements [62] (Figure 3). The device housed an inbuilt motor enabling haptic feedback and wireless communication with a computer tablet on which there were a suite of UL exercise games (GripAble app). Once participants selected an activity, the app provided instructions to guide the user. Participants were trained to use the system by an occupational therapist in a single session, issued with a standardized user manual and used the system for the remainder of their in-hospital admission. The occupational therapist rated each user’s performance in engaging with the intervention (usability) using a 4-point rating scale based on the Barthel index (BI). This enabled us to understand intervention usability and also to recommend “conditions of use” for participants (independent, modified independence, assistance, or unable). The occupational therapist also used clinical judgment to advise participants on facilitating conditions to enhance intervention performance (such as pillow support of the UL, timetabling practice, or hands-on assistance from a relative, friend, or informal caregiver) where appropriate. Participants were encouraged to use the system “as much as possible” as an adjunct to conventional therapy with all intervention advice provided using a standardized script. Participants were not prompted or supervised in use of the device during the intervention period, although they could receive assistance from relatives, friends, or informal caregivers. Participants were reviewed weekly by the research team to screen for technical issues with the intervention or identify additional user support needs. Adverse events were monitored by the treating clinical teams or self-reported by participants.

### Measures

#### Participant Characteristics

The following demographic and clinical features were recorded on study entry: age, sex, prior technology exposure (prior use of and familiarity with a smartphone, tablet, laptop, or computer, as self-reported by participants), Edinburgh Handedness Scale, time (in days) since stroke at enrollment, stroke type (ischemic or hemorrhagic), stroke severity (National Institute of Health Stroke Scale), UL impairment severity (Fugl Meyer-Upper Extremity Assessment), cognition (Montreal Cognitive Assessment), premorbid functional status (modified Rankin Scale), poststroke functional independence status (BI), mood (Hospital Anxiety and Depression Scale), fatigue (Fatigue Severity Scale), and pain (Faces Pain Rating Scale).

#### User Performance (Usability)

User performance (usability) was rated by the occupational therapist at participant enrollment or intervention setup. A 4-point scale was defined using the BI performance classification; users were scored as 4, independent; 3, requiring support for setup only (modified independence), 2, requiring supervision and support (assistance), or 1, unable to use meaningfully (unable). User performance ratings were made based on the following device functionalities: physical set up, turning on, accessing the activity platform, selecting and executing exercise software, executing the physical exercise requirements, and device charging. Final ratings were based on the lowest rating allocated for any domain of device functionality. In the context of this work, other more commonly used scales, such as the system usability scale, did not align with the features and mechanisms of this technology, the context in which it was used, and the data required to inform the intervention. Devising a custom scale enabled us to identify key functionalities associated with effective use of the device. Adopting the taxonomy of the BI enabled clear categorization of the user performance and indicated associated user support needs while also facilitating communication of user performance and needs in a language accessible to clinicians, service users, and family members or informal caregivers.

#### Technology Acceptability

**Figure 4:**
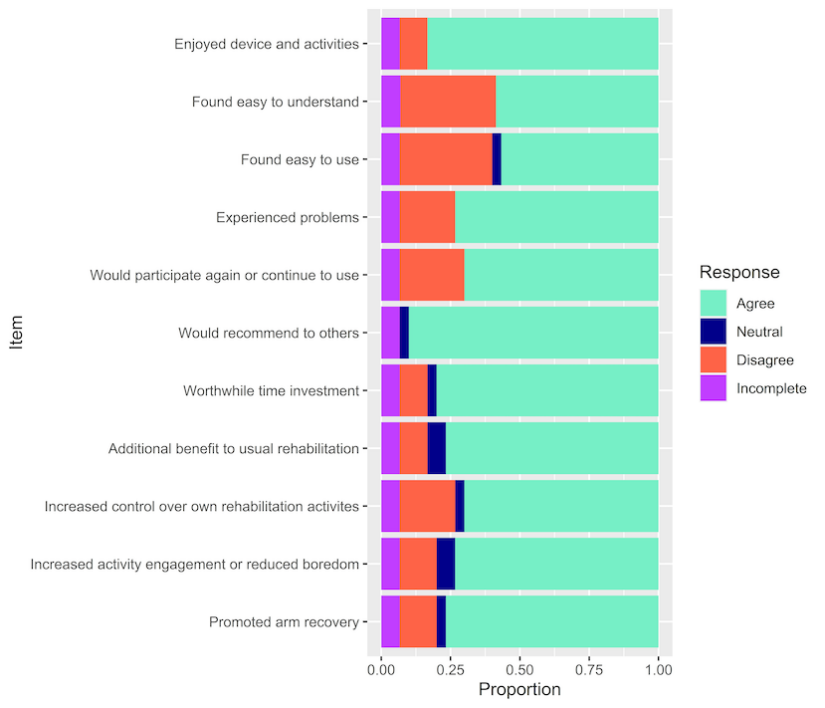
Technology acceptance survey responses.

An 11-item survey based on the TAM was adapted from available measures [51] (see Figure 4 for survey items) and administered at the study end point. Items measured included perceived usefulness (n=5 items), intentions to use (n=2 items), and perceived ease of use (n=4 items). Participants indicated their level of agreement with each item on a 3-point Likert scale (“disagree,” “neutral,” and “agree”). Participants’ comments or supporting statements in the context of their technology acceptance ratings were recorded and used as a contextual aid; no formal qualitative analysis was undertaken.

#### Technology Adoption

Adherence, defined as the active time (minutes) on a task each day (repetitive UL training or interactive gaming), was used as a surrogate measure for technology adoption [63]. Adherence was measured by (1) self-reported session times and (2) digital time-on-task recorded by the device. These measures were strongly correlated (intraclass correlation coefficient for absolute agreement *r*=0.87; *P*<.001). Self-reported times were 14.5% (IQR −0.06% to 20.9%) greater than electronic logs, since the former includes preparatory and rest periods and corresponds more closely to “time scheduled for therapy” as conventionally reported in rehabilitation studies [63,64].

### Data Analysis

Data analysis was performed with R (version 4.0.2; R Foundation for Statistical Computing) and RStudio (version 1.3.1093; Posit, PBC). Baseline clinical and demographic variables and questionnaire responses were organized into a single data matrix. Questionnaire responses were coded numerically (–1=“disagree,” 0=“neutral,” and 1=“agree”). Missing data were imputed using k-nearest neighbor imputation (k=3) [65]; imputation was performed with the caret library. Scores summarizing overall technology acceptance, perceived usefulness, intent to use, and ease of use were defined as per the formulae defined in Table 1, whereby questionnaire responses are coded numerically (–1=“disagree,” 0=“neutral,” and 1=“agree”) and combined as per the corresponding formula to generate scores.

**Table 1:** Scores summarizing overall technology acceptance, perceived usefulness, intent to use, and ease of use.

Score	Formula	Score range
Overall technology acceptance	“Promoted arm recovery” + “increased activity engagement or reduced boredom” + “increased control over own rehabilitation activities” + “additional benefit to usual rehabilitation” + “worthwhile time investment” + “would recommend to others” + “would participate again or continue to use” − “experienced problems” + “found easy to use” + “found easy to understand” + “enjoyed device and activities”	−11 to 11
Perceived usefulness	“Promoted arm recovery” + “increased activity engagement or reduced boredom” + “increased control over own rehabilitation activities” + “additional benefit to usual rehabilitation” + “worthwhile time investment”	−5 to 5
Intent to use	“Would recommend to others” + “would participate again or continue to use”	−2 to 2
Ease of use	“Found easy to use” + “found easy to understand” + “enjoyed device and activities” − “experienced problems”	−4 to 4

To assess clinical determinants of technology acceptance, bivariate correlations were measured between baseline participant characteristics (age, prior technology exposure, stroke severity, cognition, and UL impairment severity) and clinical outcomes (overall technology acceptance rating and intervention adherence). These variables were selected based on clinical reasoning and existing literature in the field indicating precedent [48]. Associations between integer variables were evaluated using 2-sided Spearman correlation tests. Bivariate associations between binary and integer variables were measured using the 2-sided Wilcoxon rank sum test. *P* values were adjusted for multiple hypothesis testing using the Holm method [66].

## Results

### Sample Characteristics

In total, 30 participants were recruited over 3 months, with 29 completing the intervention. One participant was withdrawn by the research team due to medical complications unrelated to research participation. The median enrollment duration was 8 (IQR 5-14) days. Sample characteristics and data collected are summarized in Table 2.

**Table 2:** Participant characteristics (n=30).

Variable			Value		Complete, n	
Age (years), mean (SD)			70.3 (11.9)		30	
**Sex, n**						30
	Female	16				
	Male	14				
**Stroke subtype, n**						30
	Hemorrhagic	8				
	Ischemic	22				
NIHSS,^a^ mean score (SD)			8 (4.4)		30	
Time since stroke (days), mean (SD)			11.1 (8.1)		30	
MOCA,^b^ mean score (SD)			19.9 (5.5)		24	
BI,^c^ mean value (SD)			47.1 (19.4)		29	
FM-UE,^d^ mean score (SD)			33.1 (16)		28	
FSS,^e^ mean score (SD)			5 (1.3)		29	
FPRS,^f^ mean score (SD)			1.5 (2.6)		29	
**HADS,^g^ mean score (SD)**						26
	Depression	6.7 (4.1)				
	Anxiety	5.6 (3.8)				
**Prior technology exposure, n**						30
	No	13				
	Yes	17				
Reported daily activity (minutes), mean (SD)			26 (12.1)		20	
Usability, mean score (SD)			1.6 (1)		29	

^a^NIHSS: National Institute of Health Stroke Scale.

^b^MOCA: Montreal Cognitive Assessment.

^c^BI: Barthel index.

^d^FM-UE: Fugl Meyer-Upper Extremity Assessment.

^e^FSS: Fatigue Severity Scale.

^f^FPRS: Faces Pain Rating Scale.

^g^HADS: Hospital Anxiety and Depression Scale.

### User Performance (Usability)

The technology was usable for 26 of 30 participants (87%). The remaining 4 participants (13%) were unable to use the device with their affected UL due to the severity of motor impairment (absence of voluntary finger extension or 0/5 on the Oxford Rating Scale [Medical Research Council Manual Muscle Testing Scale]). Motor weakness was monitored throughout enrollment for these 4 participants and remained unchanged. User performance varied; 7 participants were fully independent with all aspects of the technology use (device retrieval, setup, and self-directed training), 9 participants achieved modified independence (required only physical setup to use the system often due to restricted mobility), and 8 participants required assistance (supervision or support) to complete training sessions due to combined physical and cognitive impairments.

### Acceptability

The overall technology acceptance rating was 68% (95% CI 56%-79%). TAM subcategories were also explored independently. In total, 58% of respondents perceived that the device was easy to use (4 items), 86% reported an intent to use (2 items), and 77% perceived that the device was useful (5 items). Individual item responses are summarized in Figure 4.

### Adoption or Adherence

Participants (n=20) engaged with the device for a median of 26 (SD 12.1) minutes of training daily (Table 2), increasing the conventional UL training dose (25 minutes) by 2-fold [60].

### Interactions or Associations Between Variables

National Institute of Health Stroke Scale (global stroke severity) correlated positively with overall technology acceptance rating (ρ=−0.56; 95% CI −0.79 to −0.22; *P*=.007). No statistically significant correlations were observed between technology acceptance and participants’ age, prior technology exposure, Montreal Cognitive Assessment score, or Fugl Meyer-Upper Extremity Assessment score. Table 3 shows a full summary of participant variables and technology acceptance.

Lastly, associations of technology adoption with technology usability and technology acceptance variables were examined. Technology adoption (intervention adherence) correlated positively with user performance (usability: ρ=0.55; 95% CI 0.23-0.75; *P*=.007) and perceived ease of use (ρ=0.46; 95% CI 0.10-0.74; *P*=.02) as well as perceived usefulness (ρ=0.42; 95% CI 0.09-0.68; *P=*.03). No significant correlation was observed between participants’ self-reported intent to use the technology and intervention adherence during the trial period. Table 4 shows a full summary of correlations among intervention adherence, technology usability, and acceptability variables.

**Table 3:** Correlations between participant variables and technology acceptance.

Method	Predictor	Outcome	Result, ρ (95% CI)	Adjusted *P* value
Spearman	Age	Acceptance	0.04 (−0.40 to 0.44)	.85
Wilcoxon rank sum	Prior technology exposure	Acceptance	0.00 (−1.00 to 3.00)	.73
Spearman	NIHSS^b^	Acceptance	−0.56 (−0.79 to −0.22)	.007
Spearman	MOCA^c^	Acceptance	0.20 (−0.14 to 0.52)	.50
Spearman	FM-UE^d^	Acceptance	0.39 (0.00 to 0.66)	.08

^a^Location difference.

^b^FM-UE: Fugl Meyer-Upper Extremity Assessment.

^c^MOCA: Montreal Cognitive Assessment.

^d^FM-UE: Fugl Meyer-Upper Extremity Assessment.

**Table 4:** Correlations among intervention adherence, technology usability, and acceptability variables.

Method	Predictor	Outcome	Result ρ (95% CI)	Adjusted *P* value
Spearman	Usability	Intervention adherence	0.55 (0.23 to 0.75)	.007
Spearman	Perceived usefulness	Intervention adherence	0.42 (0.09 to 0.68)	.03
Spearman	Intent to use	Intervention adherence	0.25 (−0.09 to 0.54)	.18
Spearman	Ease of use	Intervention adherence	0.46 (0.10 to 0.74)	.02

## Discussion

### Principal Findings

This self-directed, technology-facilitated intervention was broadly usable and acceptable within this study cohort. Stroke severity correlated negatively with technology acceptance; those participants with the most severe stroke reported lower acceptability ratings across all domains. Participants achieved an average UL training dose of 26 minutes daily as an adjunct to conventional face-to-face UL rehabilitation. This adjunctive experimental training dose exceeded the conventional care dose typically observed in subacute stroke rehabilitation settings [64]. Technology adoption positively correlated with technology usability, perceived ease of use, and perceived usefulness, indicating that the usability of technology, as well as the effort associated with using the technology, influenced actual use. Furthermore, our findings suggest that perceived usefulness of technology, in this case the extent to which participants associated the technology with UL rehabilitation and recovery, influenced adoption. A strength of this study is the broad sampling of participants recruited in the acute or subacute stroke recovery phase, including older adults, those with cognitive impairment, and those with moderate to severe stroke, representing cohorts frequently excluded from stroke rehabilitation research [67]. Less than half of the participants (n=13, 43%) had previously owned or used a smartphone.

Although the technology was usable for the majority of participants, many required facilitating conditions to optimize their participation, highlighting the importance of assessing and addressing individual user needs. Clinical adoption of rehabilitation technologies may be improved by enhancing usability and acceptability. This may be achieved through design optimization, education, and user support, targeting the domains of usability, perceived ease of use, and perceived usefulness. In this study, a positive association was observed between perceived usefulness of technology and its adoption, presenting a promising avenue to improve engagement. A robust clinical evidence base may enhance perceived usefulness of rehabilitation technologies among stakeholders. Thus far, systematic reviews and meta-analyses have found evidence in the domain of technology-facilitated UL interventions after stroke to be insufficient or of low quality, leaving limited scope for interpreting the efficacy of such interventions [17,48,68,69] and thus restricting the extent to which clinical guidelines or individual clinicians may advocate for adoption.

This study examined a stroke rehabilitation intervention focusing on interactive gaming and nonimmersive virtual reality with a target function to achieve repetitive, task-specific UL training to promote UL motor recovery. We observed that participants with the most severe UL impairment showed a trend toward lower technology acceptance ratings. In this sense, patient characteristics can be linked with specific technology characteristics (the mechanism and target function, that is, repetitive UL training for UL recovery). Rehabilitation technology is often discussed with ambiguity; there is a lack of consensus on the taxonomy, classification, and categorization of technology. This may lead to barriers in interpreting the efficacy and applications of technology among target users. Individual technologies comprising unique mechanisms and target functions are likely to benefit from individual evaluation, incorporating the relevant user cohort to identify important interactions between user characteristics and outcomes in usability, acceptability, and adoption as well as clinical efficacy. Thorough reporting of technology subtypes and participant subgroups may advance clinical translation. The use of a framework for describing and categorizing rehabilitation technologies, and indeed digital health technologies more broadly, would likely enhance reporting standards.

### Limitations

Although this study population was heterogeneous in terms of age, sex, and clinical characteristics, it represented a single institution; future work will incorporate a multicenter design. Imputation may have biased associations where data missingness patterns were nonrandom, although multivariate imputation was used to minimize this bias. The power of our analysis was limited by the sample size—consequently, some real effects may have failed to generate statistically significant associations. The sample size was kept intentionally small to allow for feasibility testing in this instance, and while this addressed the current aims, a larger sample size will be recruited in a planned subsequent trial (ClinicalTrials.gov NCT04475692). As an observational study, findings are subject to the limitation that observed correlations do not necessarily imply causal relationships.

In the TAM survey, neutral responses were limited to questions that required a hypothetical comparison to an experience without rehabilitation technology (ie, conventional rehabilitation). The cognitive demands of such theoretical comparisons likely exceed those of questions interrogating the participants’ own experience. All respondents to the nonhypothetical questions “enjoyed device and activities,” “found easy to understand,” “experienced problems,” and “would participate again or continue to use” chose to agree or disagree rather than remain neutral. This observation may guide future survey development to improve participant engagement and response reliability. A further limitation of the TAM survey used here is that questions were largely unidirectional; inverting questions may have reduced the risk of positive response bias.

### Future Work

Findings suggest that technology acceptance and subsequently adoption negatively correlate with stroke severity in this instance. Identifying interventions for severe stroke is a key clinical, academic, and patient priority [70], a focus for future work may be on adapting technology or intervention design to enhance acceptability and adoption for those with the most severe poststroke impairments.

Technology adoption is a complex and dynamic process. We implemented a postintervention TAM survey only; administering both pre- and postintervention surveys may support our understanding of the mechanisms of technology adoption as well as mediating conditions. Several authors report significant changes in technology acceptance among users over time or in line with specific facilitating conditions (eg, social support, peer support, increased availability and frequency of training, system upgrades) [23]. Furthermore, perseverance with technology-facilitated interventions is anticipated to change over the intervention timespan [48]; understanding factors that influence the long-term adoption of rehabilitation technologies for stroke survivors will form an important aspect of future research (ClinicalTrials.gov NCT04475692).

Closed questionnaires and quantitative data collection allowed us to examine specific and tangible aspects of technology usability, acceptability, and adoption along with clinical and demographic variables; richer themes and context may be derived from a mixed methods exploration, encompassing the broader spectrum of participants’ experiences and feelings.

Finally, the adoption of health technology hinges upon multiple stakeholders and may in a large part be determined by technology usability and acceptability among clinicians [19]; this is echoed in Health Education England’s recent development of a digital competency framework for National Health Service staff [71]. In the context of this self-directed intervention, we focused on user experience from the perspective of the patient; further work may explore acceptance among broader stakeholders, including clinicians and caregivers, who play a pivotal role in supporting self-management in this setting.

### Conclusions

In an age of digitalized health care, technology usability and acceptability represent increasingly important determinants of health outcomes [9,72,73]. We explored the adoption of a low-cost (<£1000; US $1283) rehabilitation technology used in a self-directed context within a heterogeneous cohort of stroke survivors. To our knowledge, this is the first study to concurrently examine technology usability, acceptability, and adoption in this context and evaluate the influence of stroke survivor characteristics. The technology was usable and acceptable to the majority of participants and greatly supplemented conventional rehabilitation provisions. We have presented a robust analysis identifying associations between stroke survivor characteristics, technology usability, acceptability, and adoption. Our findings provide insights that will inform intervention planning and implementation, emphasize the need for specificity when reporting digital health interventions, and reiterate the importance of a holistic and person-centered approach to optimize the translation of technologies into clinical practice.

## Appendix

Multimedia Appendix 1 Stroke upper limb rehabilitation technology adoption logic model.

## Abbreviations

BI: Barthel index
TAM: technology acceptance model
UL: upper limb

## Data Availability

The data sets generated and analyzed during this study are available from the corresponding author on reasonable request.
