# Supplementary material for: Examining Usability, Acceptability, and Adoption of a Self-Directed, Technology-Based Intervention for Upper Limb Rehabilitation After Stroke: Cohort Study
Source: JMIR Rehabil Assist Technol. 2023 Aug 21;10:e45993. doi: 10.2196/45993 (PMC10477927; doi:10.2196/45993)
Supplement: Multimedia Appendix 1 [file rehab_v10i1e45993_app1.docx]

**Multimedia Appendix 1.** Stroke upper limb rehabilitation technology adoption logic model.

**User/technology variables**

- Age
- Gender
- Prior technology exposure
- Symbolism or images associated with technology(subjective norms)
- Perception of results (result demonstrability)
- Perception of relevence to current situation/role
- Self-efficacy in the use of technology
- Perceptions of external control
- Perceived enjoyment
- Technology user anxiety
- Technology user playfulness
- Social circumstances (availability of social, emotional & pragmatic support, social roles and responsibilities)
- Cognition
- Mood
- Motivation
- Energy/fatigue
- Sensory awareness (tactile/proprioceptive)
- Pain
- Physical ability (UL strength and range of motion/mobility/seating tolerance or requirements)
- Global burden of post-stroke impairments (stroke severity)
- Functional independence status
- Objective usability; technology accessibility/ease and efficiency of use (functionalities, user interface, set-up time)
- Size/ergonomics of technology
- Availability of technology
- Affordability of technology
- Technology to user fit (“just right challenge”)
- User performance feedback and tracking
- User performance/capability in engaging with the technology
- User education (rationale for intervention)
- User training (use of the technology)
- User support
- Reliability of technology (battery performance, software/hardware malfunction, durability)
- Safety of technology/intervention

**Perceived usefulness**

Does the participant consider the device to be part of their rehabilitation? Do they perceive it to have a benefit in the context of their UL recovery?

**Technology acceptance**

Beliefs and feelings about/ attitude towards the technology

**Intent to use**

Behavioural intention: expressed intent to use the technology

**Actual use**

Technology adoption/intervention adherence; self-directed engagement in UL training

**Perceived ease of use**

Does the participant perceive engaging with the device/system to be difficult or effortful?
